# Suprainguinal ectopic scrotum: a case report

**DOI:** 10.3389/fsurg.2026.1807545

**Published:** 2026-05-08

**Authors:** Xiaozhu Chen, Weiwu Pan, Weite Qian, Liguang Xia

**Affiliations:** Department of Pediatric Urology, Second Affiliated Hospital and Yuying Children’s Hospital of Wenzhou Medical University, Wenzhou, China

**Keywords:** case report, ectopic scrotum, scrotoplasty, solitary kidney, testicular repositioning

## Abstract

Ectopic scrotum is a type of congenital scrotal anomaly, most cases can be diagnosed in infancy. Various surgical management strategies of ectopic scrotum has been reported in the literature, but none has been proven to be superior. We report a case of suprainguinal ectopic scrotum in a 1-year-old boy with an abnormal sac of scrotal skin in the suprainguinal region since birth. We performed a one-stage scrotoplasty and testicular repositioning, achieving a satisfactory outcome.

## Introduction

Ectopic scrotum (ES) refers to the abnormal anatomical positioning of a hemiscrotum along the inguinal canal, which is a rare form of congenital scrotal anomaly. ES most frequently occurs in the inguinal, infrainguinal, suprainguinal and perineal regions, with the suprainguinal variant being the most common (1). While diagnosis is typically made by physical examination in infancy, there is no universal consensus on its surgical management, with reported techniques varying considerably. We report a rare case of suprainguinal ectopic scrotum complicated with a solitary kidney, which was successfully treated with one-stage scrotoplasty and testicular repositioning.

## Case presentation

**Figure 1:**
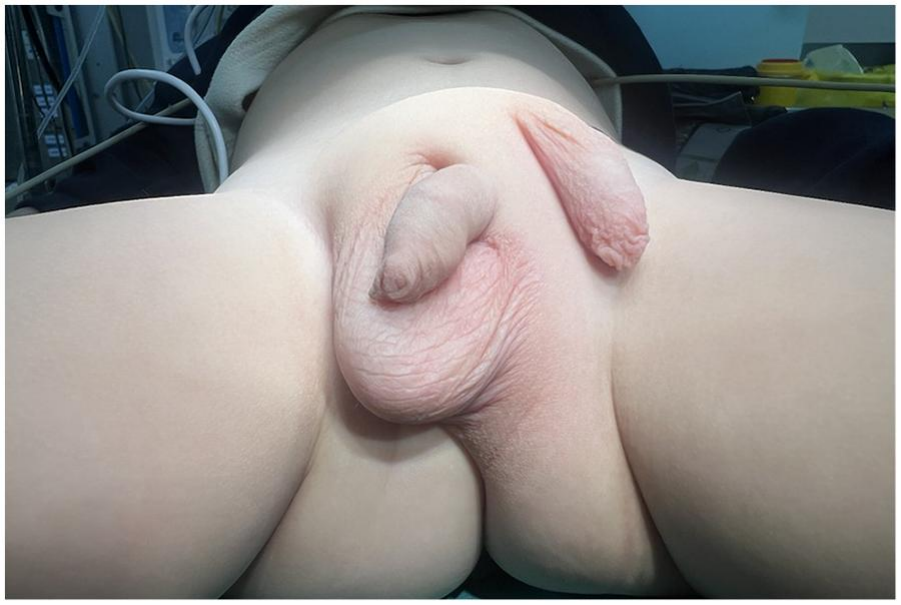
Preoperative appearance.

A 1-year-old boy was admitted to our hospital due to an abnormal sac of scrotal skin in the suprainguinal region, which had been present since birth (Figure 1). He was the only child of his family, and the mother denied exposure to any medications, toxins, or harmful substances during pregnancy. No family history of similar abnormalities was identified.

A complete physical examination was performed, and the right hemiscrotum was observed to be in its normal anatomical position housing a testis of normal size. The left hemiscrotum was located in the left suprainguinal region, containing a testis which was similar in size to the right one. The penis was normally developed, while the scrotal raphe was completed absent. Ultrasound examination of abdomen showed a solitary right kidney, and no other congenital anomalies were found in the genitourinary or other systems.

**Figure 2:**
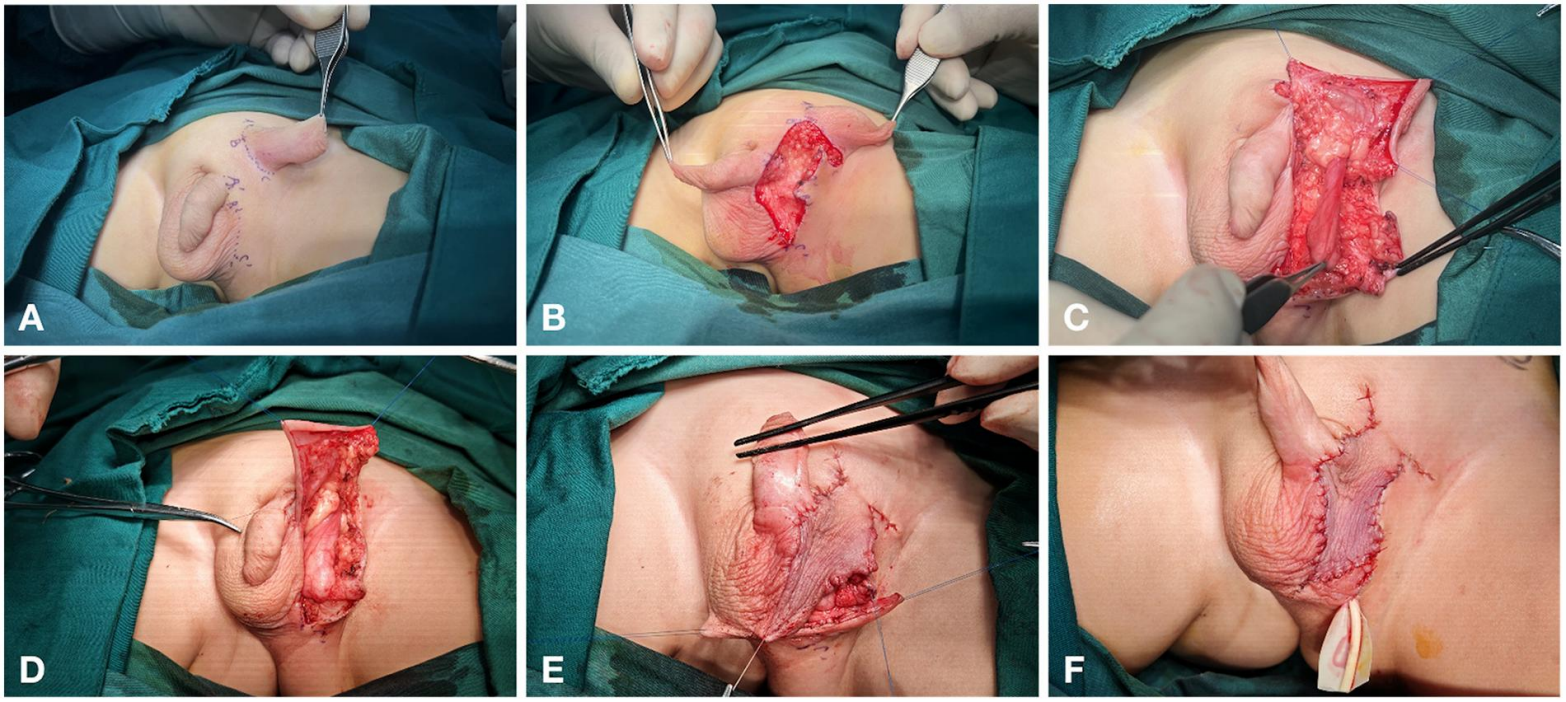
Surgical procedure: **(A)** designed the incision. **(B)** made an incision at the left inguinal region. **(C)** separated the testis and spermatic cord. **(D)** fixed the testis. **(E)** trimmed the scrotal flap. **(F)** placed a drainage strip.

**Figure 3:**
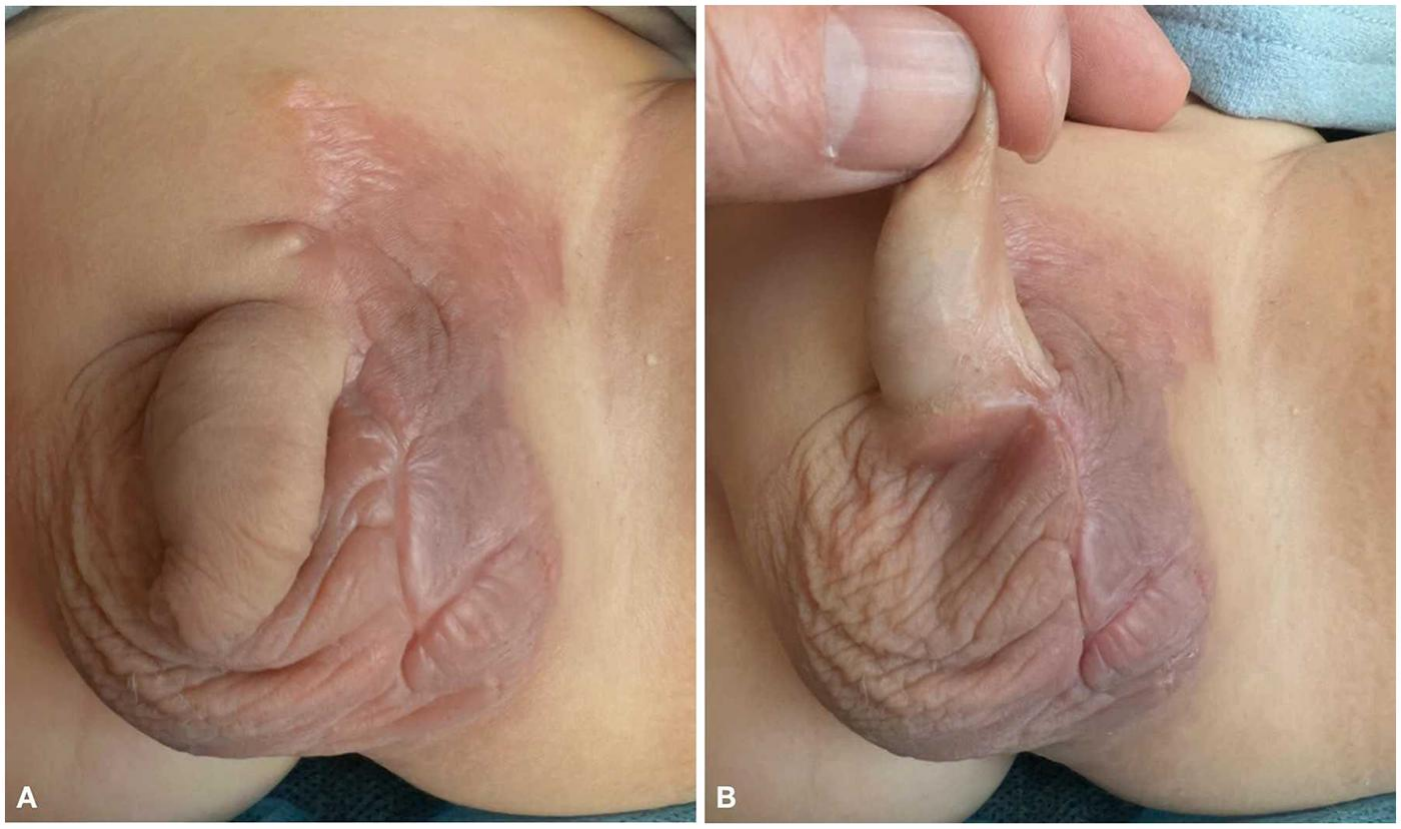
1-month postoperative appearance.

A one-stage surgical procedure was performed for the treatment of ectopic scrotum. The procedure began with an incision made on the medial aspect of the ectopic scrotum in the left inguinal region, extending along the left root of the penis to the distal end of the native scrotal raphe. The testis in the sac was carefully dissected, with preservation of its vascular and spermatic cord, to enable tension-free descent and secure fixation at the original anatomical position. During the operation, the processus vaginalis was found to be closed, which required no additional intervention. Then, the left scrotum was reconstructed using an inguinal rotational scrotal flap, with a drainage strip placed at the base of the reconstructed scrotum (Figure 2). During the subsequent follow-up, the scrotal flap survived well with a satisfactory cosmetic appearance. The repositioned testis remained in the anatomical scrotal position without retraction, and its development was comparable to that of the contralateral side (Figure 3).

## Discussion

Ectopic scrotum is one of the congenital scrotal anomalies, with three other subtypes including penoscrotal transposition, bifid scrotum and accessory scrotum (2). ES mainly occurs in inguinal, suprainguinal, infrainguinal and perineal regions (3), however, the exact embryological etiology of this condition remains unclear.

Scrotal development initiates during the fourth week of gestation, with paired labioscrotal swellings emerging in the inguinal region. By the twelfth week of gestation, these swellings migrate medially and inferiorly, and merge beneath the penis to form the scrotum and scrotal raphe (4). During the fifth to sixth weeks of gestation, the precursor of gubernaculum develops, originating from the urogenital ridge and extending towards the labioscrotal swellings. The distal end of the gubernaculum attaches to the base of the scrotum. Between the eighth and fifteenth week of gestation, the gubernaculum gradually elongates, facilitating the descent of the testis towards the inguinal region. During the third trimester of gestation, the gubernaculum enlarges and directs the testis into the scrotum (5). Therefore, the labioscrotal fold's migration or differential growth, or a developmental defect of the gubernaculum, may contribute to ES. On the other hand, it has been reported that the direct mechanical compression caused by the contralateral heel could be a potential cause of ES. In such cases, there is typically a disproportion in size between fetus and chorionic sac or oligohydramnios, which leads to an abnormal limb position (6, 7).

Ectopic scrotum often associate with other congenital anomalies, including inguinal hernia, cryptorchidism, imperforate anus, bladder exstrophy and VACTERL association (3). There are also cases with no associated congenital anomalies reported in the literature. Approximately 70% of patients with suprainguinal ectopic scrotum are complicated by ipsilateral upper tract anomalies (8). In our case, there is a solitary right kidney showed in ultrasound examination. Both the scrotum and kidney originate from the urogenital ridge of the embryonic mesoderm, and their development is highly spatiotemporally coordinated. Thus, abnormal differentiation of the urogenital ridge often results in anomalies of the scrotum and kidney. Seth has demonstrated that WNT4 deficiency can lead to abnormal development of the gubernaculum (9), while Vivante has indicated that WNT4 variants may impair renal development (10). Given the role of WNT4 in both gubernacular development and renal morphogenesis, a variant in this pathway could theoretically explain the concurrent anomalies in our patient, though this remains speculative in the absence of genetic analysis.

The clinical diagnosis of ectopic scrotum is not difficult. Given that it is often associated with other congenital anomalies, a comprehensive physical and auxiliary examinations are crucial for patients with ectopic scrotum. It is important to distinguish ectopic scrotum from other scrotal anomalies, particularly accessory scrotum, which does not contain testis and can be managed with simple excision (11). While, the ectopic scrotum usually need a scrotoplasty and testicular repositioning.

Most cases of ectopic scrotum can be identified during the neonatal period, though a few cases have also been reported in adults, such as an 18-year-old male who complained about lower abdominal and perineal pain when exercise (12). Ectopic scrotum is not only a cosmetic issue, but also potential adverse effects on testicular development. Animal studies have revealed impaired testicular development and spermatogenesis in rats with ectopic scrotum (13). Therefore, timely surgical intervention is of particular importance for ectopic scrotum, with the optimal timing for surgery reported to be six to twelve months of age (8).

A broad spectrum of surgical techniques has been reported in the literature. Guha corrected the ES by resecting the ectopic scrotal skin and fixing the testis in the contralateral scrotum (14). Daniel proposed a staged surgical approach, with rotation flap scrotoplasty performed in the first stage and orchiopexy conducted six months later (15). Alyamani utilized the double opposing transposition Z-plasty flap technique (16). Conversely, Sobral adopted an inverted Y shaped incision to correct the ectopic scrotum and penis in a single procedure (17). In our case, ectopic scrotal tissue was used to create scrotal flaps for scrotal reconstruction, yielding a more esthetically pleasing, fuller appearance. Simultaneously, the absent neoscrotal raphe was surgically constructed. Given the patient's tender age, prolonged follow-up is required to assess the development and function of the left testis. Yet, there is no consensus on which surgical approach is superior. A personalized treatment plan should be established based on a comprehensive consideration of the anatomical positioning of the ectopic scrotum and testes, the condition of the scrotal flaps, any associated comorbidities, as well as the operating surgeon's clinical preferences.

In conclusion, ectopic scrotum is clinically rare and often accompanied by other congenital malformations, with its specific etiology remaining unclear. Different surgical management strategies for ectopic scrotum have been reported in the literature, we achieved satisfactory outcomes by performing one-stage scrotoplasty and orchiopexy using a rotation flap.

## Data Availability

The raw data supporting the conclusions of this article will be made available by the authors, without undue reservation.
